# Modelling the multiple anatomical site transmission of *Mycoplasma genitalium* among men who have sex with men in Australia

**DOI:** 10.1038/s41598-021-90627-3

**Published:** 2021-05-27

**Authors:** Xianglong Xu, Catriona S. Bradshaw, Eric P. F. Chow, Jason J. Ong, Jane S. Hocking, Christopher K. Fairley, Lei Zhang

**Affiliations:** 1 China Australia Joint Research Center for Infectious Diseases, School of Public Health, Xi’an Jiaotong University Health Science Centre, Xi’an, 710061 Shaanxi People’s Republic of China; 2 Melbourne Sexual Health Centre, Alfred Health, Melbourne, Australia; 3 Central Clinical School, Faculty of Medicine, Nursing and Health Sciences, Monash University, Melbourne, Australia; 4 Centre for Epidemiology and Biostatistics, Melbourne School of Population and Global Health, The University of Melbourne, Melbourne, Australia; 5 Department of Epidemiology and Biostatistics, College of Public Health, Zhengzhou University, Zhengzhou, Henan People’s Republic of China

**Keywords:** Bacterial infection, Epidemiology, Preventive medicine, Applied mathematics

## Abstract

*Mycoplasma genitalium* (*M. genitalium*) is a recently recognised and important sexually transmitted infection among men who have sex with men (MSM). The role of oral sex, rimming, and kissing on *M. genitalium* transmission in MSM is unclear. We created four deterministic susceptible-infectious-susceptible epidemic models to examine the role that different sexual behaviours play in transmitting *M. genitalium* at the oropharynx, urethra anorectum among men who have sex with men in Australia. Our results suggest that oral and anal sex without other sexual practices (model 1) replicate well single site infection at the oropharynx, urethra and anorectum and also multi-site infection. If kissing or rimming are added to model 1 (i.e., model 2–4) no substantial improvements in the calibration of the models occur. Model 1 estimates that 3.4% of infections occur at the oropharynx, 34.8% at the urethra and 61.8% at the anorectum. Model 1 also estimates that the proportion of incident *M. genitalium* transmitted by anal sex was 82.4%, and by oral sex was about 17.6%. Our findings could provide an enhanced understanding of *M. genitalium* transmission in MSM, thus providing insights into what sexual practices contribute most to transmission.

## Introduction

*Mycoplasma genitalium* (*M. genitalium*) is a recently recognised sexually transmitted infection (STI) that is becoming a common STI among men who have sex with men (MSM) globally^1,2^. Furthermore, *M. genitalium* is rapidly developing substantial antibiotic resistance^3,4^ and has become a difficult STI to treat^2,5,6^. In this context, the prevention of *M. genitalium* infection is an important strategy, although preventing it will be challenging given the marked reduction in condom use that has occurred with biomedical interventions for HIV prevention such as pre-exposure prophylaxis^7^. Developing interventions for the prevention of *M. genitalium* in MSM will require a detailed understanding of *M. genitalium* transmission routes. Current empirical studies have primarily addressed transmission by anal sex.

*Mycoplasma genitalium* and chlamydia commonly occurs at the anorectum and urethra, although oropharyngeal infection also uncommonly occurs. In contrast, gonorrhea could commonly occur at the anorectum and oropharynx. A meta-analysis summarised the prevalence of *M. genitalium* among at different sites in MSM, with 1.0% (95% CI 0.0–5.1%) of men having an infection at the oropharynx, 5.0% (95% CI 3.5–6.8%) at the urethra, and 6.2% (95% CI 4.6–8.1%) at the anorectum^1^. Another meta-analysis estimated the prevalence of chlamydia and gonorrhea among individuals using pre-exposure prophylaxis^8^. The estimated prevalence at the oropharynx (chlamydia, 2.4% [95% CI 0.9–4.5%]; gonorrhea, 4.9% [95% CI 1.9–9.1%]), (chlamydia, 4.0% [95% CI 2.0–6.6%]; gonorrhea, 2.1% [95% CI 0.9–3.7%]) at the urethra, and (chlamydia, 8.5% [95% CI 6.3–11.0%]; gonorrhea, 9.3% [95% CI 4.7–15.2%]) at the anorectum^8^. Some *M. genitalium* infection uncommonly occurs at more than one site simultaneously (multi-site infection). About 1.47–2.97% of infections occur at more than one site^9,10^.

Epidemiological studies focusing on the association between sexual practices and *M. genitalium infectious* are limited and often do not include detailed information on sexual practices other than anal sex. A survey reported that always using condoms for penile-anal sex in the last three months was a protective factor for *M. genitalium* infection (OR 0.8; 95% CI 0.6–1.0)^9^. Another study of 409 MSM in Shenzhen, China, reported that condomless penile-anal intercourse in the past six months had higher odds of acquiring urethral *M. genitalium* infection (OR 4.8; 95% CI 1.4–16.5)^11^. These studies did not include all potential such as oral sex, rimming or kissing on the transmission of *M. genitalium* despite these practices having been shown to transmit other bacterial STIs such as *Neisseria gonorrhoeae* and *Chlamydia trachomatis*^12,13^.

Mathematical models can investigate different transmission routes and the plausibility of transmission between different anatomical sites, particularly when the transmission may be complex or difficult to study epidemiologically^13,14^. Investigating the potential role of different sexual practices for the transmission of *M. genitalium* in MSM using epidemiological studies is difficult because many sexual practices occur together in the same sexual encounter necessitating large numbers in studies to separate the role of each sexual practice^15^. For example, kissing, oral sex, riming, and anal sex more often occur together and are so correlated that it is virtually impossible to look at the independent contribution of different practices either statistically or through simple stratifications^16^.

We and others have created anatomical site-specific mathematical models in *Neisseria gonorrhoeae*^17–19^ and *Chlamydia trachomatis*^15^, but no mathematical models have explored the transmission of *M. genitalium* in MSM. A few mathematical models have been published in heterosexuals^20,21^, although none of these studies used anatomical site-specific models. We aimed to develop a series of anatomical site-specific mathematical models to determine what sexual practices were necessary to replicate single-site infection of *M. genitalium* at the oropharynx, anorectum and urethra and multi-site infections.

## Results

### Calibration of *M. genitalium* transmission model

**Figure 1:**
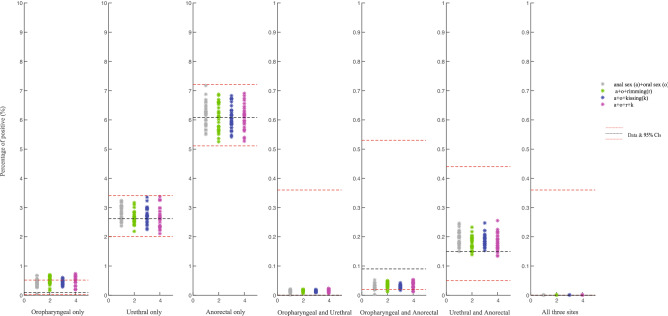
Empirical site-specific positivity data of *Mycoplasma genitalium* and the positivity data simulated by the four fitted models. The black dotted lines represent the mean empirical site-specific positivity data; The two red dotted lines indicate the 95% CIs for the mean empirical site-specific positivity data; The positivity of oropharyngeal and urethra both and infection at all three sites was zero (mean value and lower 95% CI), and therefore the dashed lines are missing; Model 1 (grey asterisk): Oral sex and anal sex only; Model 2 (green asterisk): Oral sex and anal sex and rimming only; Model 3 (blue asterisk): Oral sex and anal sex and kissing only; Model 4 (purple asterisk): Oral sex and anal sex and rimming and kissing; Oropharyngeal only: infection at the oropharynx only; Urethral only: infection at the urethra only; anorectal only: infection at the anorectum only; oropharyngeal and urethral: infection at both oropharynx and urethra; oropharyngeal and anorectal: infection at both oropharynx and anorectum; urethral and anorectal: infection at both urethra and rectum; all three sites: infection at all three anatomical sites.

Figure 1 shows the model's outputs, including estimates of the simulated prevalence of single-site and multi-site infection at the oropharynx, urethra and anorectum. Model 1 (transmission by only penile-anal sex and penile-oral sex) was able to fit the empirical prevalence data of single-site and multi-site infection at the oropharynx, urethra and anorectum (Fig. 1). When we added rimming, kissing or both to model 1 (models 2–4), we could also fit the empirical prevalence data of single-site and multi-site infection at the oropharynx, urethra and anorectum (Fig. 1).

To select the best-fitting model, we evaluated the models by generating their sum of squared errors (SSE) and compared the results of models 2–4 to model 1. Model 2 (addition of rimming only) and model 4 (addition of both rimming and kissing) demonstrated a significantly higher error in calibration to empirical data than model 1 (model 2 with an SSE of 11.51 × 10^–6^ [95% CI 3.57 × 10^–6^–14.77 × 10^–6^] vs. model 1 with an SSE of 6.43 × 10^–6^ [95% CI 3.38 × 10^–6^–7.56 × 10^–6^], p < 0.001; model 4 with an SSE of 10.04 × 10^–6^ (95% CI 1.12 × 10^–6^–12.79 × 10^–6^) vs. model 1 with an SSE of 6.43 × 10^–6^ [95% CI 3.38 × 10^–6^–7.56 × 10^–6^], p < 0.001). In contrast, model 3 (addition of kissing only) showed no significant difference from model 1 (model 3 with an SSE of 6.08 × 10^–6^ (95% CI 2.17 × 10^–6^–7.20 × 10^–6^) vs. model 1 with an SSE of 6.43 × 10^–6^ [95% CI 3.38 × 10^–6^–7.56 × 10^–6^], p = 0.406) (Supplementary Table S1). The model estimated anatomical per-act transmissibility was provided in the supplementary materials (Supplementary Fig. S1).

### Using calibrated models to estimate the incidence of *M. genitalium*

**Figure 2:**
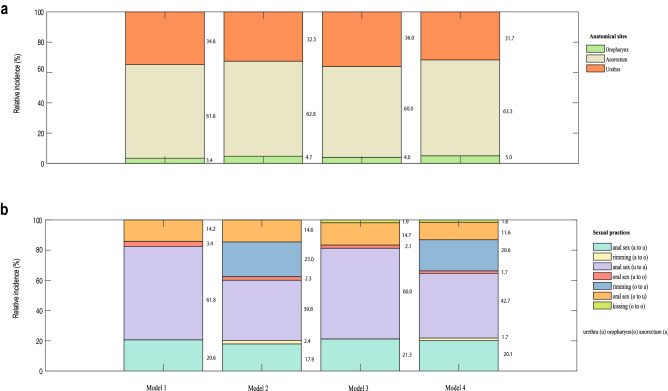
The estimated relative incidence of *Mycoplasma genitalium*. (**a**) The estimated relative incidence at the oropharynx, anorectum or urethra in MSM from the four models (%). Model 1: Only oral sex and anal sex transmit *Mycoplasma genitalium*; Model 2: Only oral sex, anal sex and rimming transmit *Mycoplasma genitalium*; Model 3: Only oral sex, anal sex and kissing transmit *Mycoplasma genitalium*; Model 4: Oral sex, anal sex, rimming, and kissing transmit *Mycoplasma genitalium*. (**b**) The estimated relative incidence caused by sexual practices in MSM from the four models (%). Model 1: Only oral sex and anal sex transmit *Mycoplasma genitalium*; Model 2: Only oral sex, anal sex and rimming transmit *Mycoplasma genitalium*; Model 3: Only oral sex, anal sex and kissing transmit *Mycoplasma genitalium*; Model 4: Oral sex, anal sex, rimming, and kissing transmit *Mycoplasma genitalium*.

We used model 1 (our best-fitting model) to explore the estimated incidence at different anatomical sites (oropharynx, urethra and anorectum) or the contribution that different sexual practices made to incident *M. genitalium* infection. Model 1 estimated that anorectal infection accounted for 61.8% of incident cases, urethral infection for 34.8% and oropharyngeal infection for 3.4% of incident cases. The proportion of incident infections that occurred at the oropharynx, anorectum or urethra in the four models is shown in Fig. 2a. We also provided person-years incidence at the oropharynx, anorectum or urethra in the supplementary materials. (Supplementary Table S2).

To explore the relative importance of sexual practices for new *M. genitalium* infection, we estimated the proportion of incident infections due to specific sexual practices in the four models, and this is shown in Fig. 2b. Our best model (Model 1) estimated that the proportion of incident *M. genitalium* infections due to penile-anal sex only was 82.4%, and penile-oral sex only was 17.6%. Even in the models that included riming and kissing, only a relatively small proportion of cases were due to these sexual practices, with kissing responsible for only 1.6–1.9% of cases (Model 3 and 4).

### Sensitivity analysis

We performed sensitivity analyses on model 1 (oral and anal sex only). The results showed that varying key model parameters (duration of infection and frequency of sexual practices) did not alter our conclusions. Model 1 still reliably replicated single-site and multi-site infections at the oropharynx, urethra, and anorectum (Supplementary Fig. S2). Furthermore, our sensitivity analyses did not significantly change the proportion of incident infections that occurred at the oropharynx (1.8–3.6%), anorectum (61.5–68.8%) or urethra (28.7–35.1%). (Supplementary Fig. S3a). Our sensitivity analyses did not significantly change the proportion of incident infections due to penile-anal sex only was 84.0–86.9% and penile-oral sex only was 13.1–16.0%. (details in the supplementary results, Supplementary Fig. S3b).

## Discussion

Our model is the first model to explore the role that different sexual practices play in transmitting *M. genitalium* to different anatomical sites in MSM. Our findings suggest that oral and anal sex alone can explain the *M. genitalium* prevalence data at the oropharynx, urethra, and rectum (either alone or in combination) without the need to invoke transmission by kissing or rimming. The inclusion of rimming or kissing did not substantially improve our model’s calibration that included only anal sex and oral sex. Our model also demonstrates that the anorectum is the most important site, followed by the urethra and that the oropharynx is relatively unimportant in the transmission of *M. genitalium* between men. Our model shows that penile-anal sex is the main contributor to new *M. genitalium* infections. Our findings suggest that effective prevention measures to control *M. genitalium* infection will need to reduce transmission by penile-anal sex, accounting for more than 80% of incident cases. Our results indicate that oral sex may be responsible for 18% of new infection cases in MSM. Our study needs to be confirmed in epidemiological studies, but our findings could provide some guidance for the future direction of *M. genitalium* studies.

Our findings suggest that incident *M. genitalium* infection is uncommon and that oropharyngeal infection may be due to penile-oral sex. We estimated that only 3.4% of incident *M. genitalium* infection occurred at the oropharynx. Our findings may help explain the observation that oropharyngeal *M. genitalium* infection is not common in MSM^1,9^. Our results may suggest that new oropharyngeal infection (3.4%) arises from urethral infection through penile-oral sex (from the urethra to oropharynx) and probably not from kissing. The estimated incidence of oropharyngeal infection is substantially lower than anal infection, consistent with oropharyngeal infection being uncommon in MSM^9,10,22^. Nevertheless, we estimate that about 40.8% (14.2%/34.8%) of new urethral infections could result from oral sex.

We also investigated the role of that oropharyngeal *M. genitalium* infection could potentially play if it were transmitted by other sexual practices other than oral sex. While models 2–4 were either more or not different from model 1, we found that between 1.7% (model 4) to 2.4% (model 2) of new oropharyngeal infection may arise from anal infection through rimming (from anorectum to oropharynx). Our model also predicted that between 1.6% (model 4) to 1.9% (model 3) of new oropharyngeal infection might arise from anal infection through kissing (from the oropharynx to oropharynx). Future empirical studies will be needed to confirm or refute the findings of our models.

Our study suggests that new *M. genitalium* infection mainly occurs at the anorectum and urethra, with 61.8% of incident cases occurring at the anorectum and 34.8% at the urethra (model 1). Our best model (model 1) also estimated that insertive penile-anal sex contributed significantly more to new infection than receptive anal sex (61.8% vs. 20.6%). Thus, preventing transmission from condomless anal sex, particularly insertive penile-anal sex, is important for preventing *M. genitalium infection* at the urethra or anorectum in MSM^11,23^. Under this context, we hope our work could encourage further empirical research to explore our estimates for the prevention of *M. genitalium* through condomless anal sex.

This study has some limitations. First, there were limited publications on the epidemiology of *M. genitalium* site-specific infection in MSM to test our models, which meant we used only three studies to calibrate our models. The proportion of MSM who had multi-site infections of *M. genitalium* was relatively low in all three studies, and therefore our estimate has wide confidence intervals. We calibrated our model to the weighted average of the prevalence to narrow confidence intervals for precise model calibration. Second, we had to make some assumptions about the parameters when published data was not available. For example, the natural history parameters for *M. genitalium* were particularly uncertain^20^, and little is known about the natural history of untreated infection^24^. We, therefore, assumed some parameters for *M. genitalium* because the natural history of *M. genitalium* is analogous to chlamydia^25^. Uncertainties in the proportion of asymptomatic urethral infection and bacterial load at various anatomical sites may affect the estimate of transmission^17^. To address this issue, we performed uncertainty and sensitivity analysis. Our sensitivity analyses showed that varying key model outcome indicators (duration of infection and frequency of sexual practices) did not alter our conclusions related to *M. genitalium* model calibration and incidence estimation. Moreover, there may be other sexual practices that we did not consider in our *M. genitalium* model.

## Methods

### Model overview

**Figure 3:**
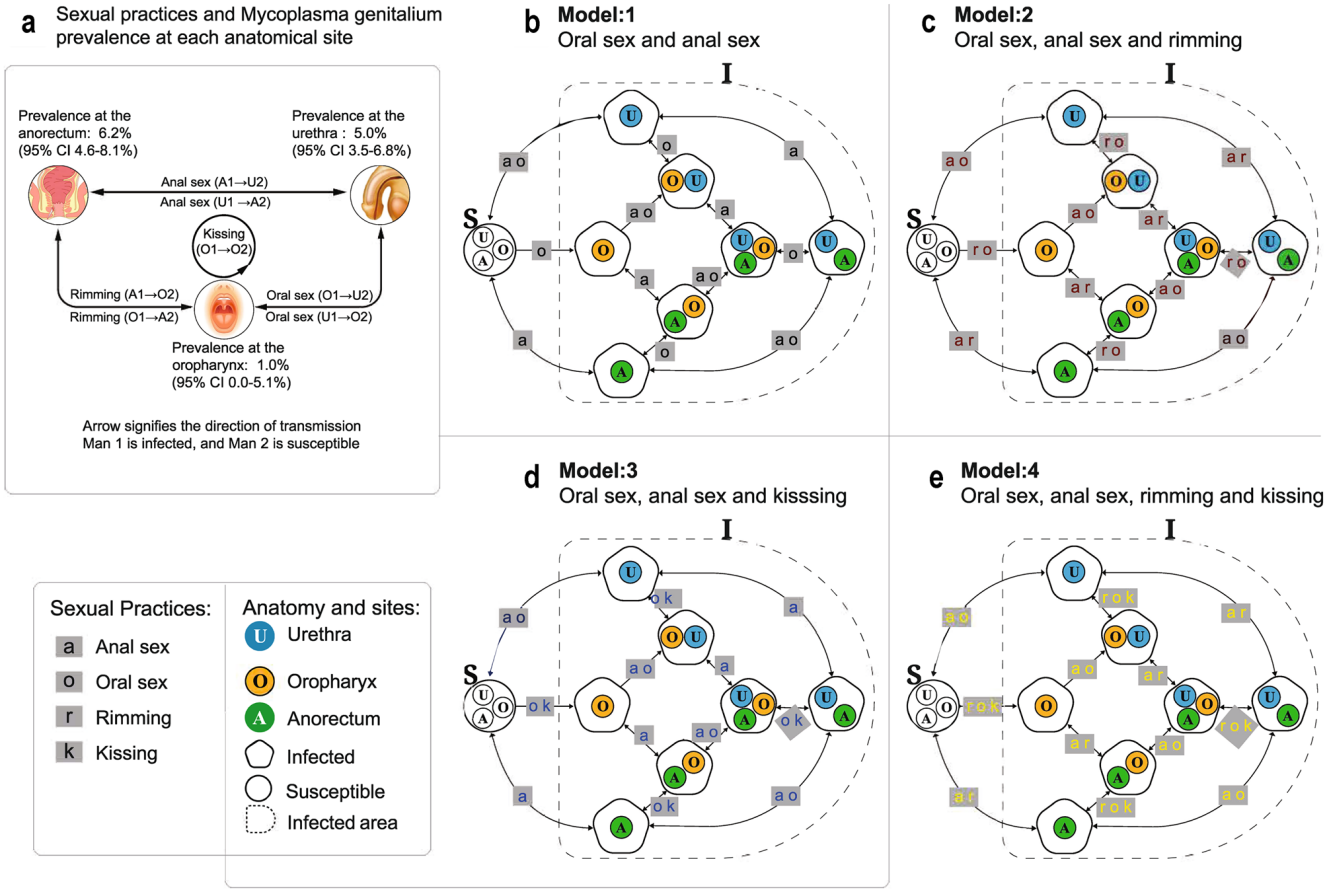
Sexual practices, site-specific prevalence, and model structure. (**a**) Sexual practices and site-specific prevalence. Man 1 is infected, and Man 2 is susceptible; U = urethra, A = anorectum, O = oropharynx; The numerical subscript number (1 or 2) refers to Man 1 or Man 2 (e.g. A1 = anorectum of man 1); Site-specific prevalence was from Sexually Transmitted Infections (2020; sextrans-2019-054310); (**b**) Model 1: Only oral sex and anal sex transmit *Mycoplasma genitalium*; (**c**) Model 2: Only oral sex, anal sex and rimming transmit *Mycoplasma genitalium*; (**d**) Model 3: Only oral sex, anal sex and kissing transmit *Mycoplasma genitalium*; (**e**) Model 4: Oral sex, anal sex, rimming, and kissing transmit *Mycoplasma genitalium*.

We constructed a compartmental model to simulate the transmission of *M. genitalium* among MSM. Since an individual can be immediately susceptible again after recovery from *M. genitalium* infection, we developed deterministic susceptible-infectious-susceptible epidemic models for *M. genitalium* transmission^26^. Our *M. genitalium* models are based on previous published anatomical site-specific models^15,17–19^. The *M. genitalium* model incorporated eight states of infection, including susceptible, single-site infection (infection at the oropharynx only, infection at the urethra only, infection at the anorectum only), and multi-site infection (infection at both oropharynx and urethra, infection at both oropharynx and anorectum, infection at both urethra and anorectum, and infection at all three anatomical sites) (Fig. 3).

In our baseline model, we included oral sex and anal sex because both have been shown to play a role in the transmission of gonorrhoea and chlamydia^12,27,28^. We then built other models by progressively adding sexual practices, such as kissing and rimming that have been shown to play a role in transmission in other STIs^12,13^, to determine what sexual practices best replicated the observed prevalence at each anatomical site (Fig. 3).

Our *M. genitalium* models included the following assumptions: (1) *M. genitalium* multi-site infection could develop in a man who is already infected at one anatomical site when he has sex with another infected partner; and (2) oropharyngeal infection attributed to sexual practices involved oropharynx site such as oral sex, rimming, and kissing.

### Model development

We established four compartmental models to test the effect of different sexual practices on the transmission of *M. genitalium* (outlined in Fig. 3). These transmission routes included: (a) penile-anal sex and penile-oral sex; (b) penile-anal sex and penile oral sex and oral-anal sex (rimming); (c) penile-anal sex and penile-oral sex and kissing; and (d) penile-anal sex and penile-oral sex and rimming and kissing. The sexual practices simulated in models 1 to 4 were demonstrated in Fig. 1. The *M. genitalium* models (models 1–4) were represented as a group of ordinary differential equations (Supplementary Table S1).

### Data sources and model parameters

Our model parameters were collected from previously published biological and behavioural data of *M. genitalium* (Supplementary Table S2). Unlike *Neisseria gonorrhoeae* and *Chlamydia trachomatis*, international guidelines do not recommend screening for *M. genitalium* at any site^1^. A recent study concluded that offering screening for *M. genitalium* to MSM could slightly reduce the prevalence and incidence but also substantially increase the selection of macrolide resistance^29^. Therefore, we did not include screening in our models. The detailed calibration procedures were provided in the Supplementary Information.

We calibrated the model to the prevalence of *M. genitalium* infections. Based on a previous systematic review and meta-analysis findings, we knew that *M. genitalium* was uncommonly detected in the oropharynx^1^. The meta-analysis collected data of oropharyngeal *M. genitalium* from seven studies. We excluded two studies from conference abstracts because of the inadequate description of the study methods. Among the remaining five studies, one study reported the required information stratified by anatomical sites^9^. Then we contacted authors from the other four studies, and two responded to us with data with stratification of multi-site infection^10,22^. Finally, there were three available studies with single-site infection and multi-site infection data. We established *M. genitalium* models with oropharyngeal infection in MSM based on these three Australia studies with multi-site infection^9,10,22^. These three studies included 2040 MSM, and the overall proportion of positive infection among those tested was 8.9% (180/2030). Based on the sample size of the included individuals studies, we calculated the weighted average of the prevalence of *M. genitalium* infections at each anatomical site (i.e. oropharynx: 0.1% [95% CI 0.0–0.5%], urethra: 2.6% [95% CI 2.0–3.4%], and anorectum: 6.1% [95% CI 5.1–7.2%), and multi-site infection (oropharynx and urethra together: 0.0% [95% CI 0.0–0.4%], oropharynx and anorectum together: 0.1% [95% CI 0.0–0.5%], urethra and anorectum together: 0.2% [95% CI 0.1–0.4%], oropharynx and urethra and anorectum together: 0.0% [95% CI 0.0–0.4%]) for model calibration (details in Supplementary Table S3).

### Model calibration and model outputs

The two key model outputs included model-estimated prevalence and incidence. We sampled the parameter space using Latin Hypercube Sampling (LHS) based on the ranges of our input parameters. We simulated 300 parameter sets using LHS as the initial points for calibration. For each set, we simulated the transmission to obtain the equilibrium prevalence at single-site infection (infection at the oropharynx only, infection at the urethra only, infection at the anorectum only), and multi-site infection (infection at both oropharynx and urethra, infection at both oropharynx and anorectum, infection at both urethra and anorectum, and infection at all three anatomical sites). We measured the calibration error by calculating the sum of squared error between the empirical infection data and the corresponding model-simulated results. We used *fmincon*, a MATLAB routine that employed a ‘trust-region-reflective’ optimisation approach, to minimise the sum of squared error (SSE) for each of the 300 simulations^30^. Out of these simulations, we sorted the simulation outputs in the descending order of SSE. The top 10% of simulations with the least SSE were regarded were used to generate the 95% confidence intervals of the model outputs. We used an independent-samples t-test to examine the differences in the SSE between two models^15,19^. Statistical significance was considered at p < 0.05. All analyses were conducted in MATLAB R2019a. The model parameters, model calibration process have been described in detail in the Supplementary S2.

We used the calibrated models to estimate *M. Genitalium* incidence. In brief, we estimated the new *M. Genitalium* infections at any given time and calculated the ratio between the number of new infections and the number of susceptible men. The study methods have been reported previously^15,17,19^. We assessed the relative incidence (proportion of incidence cases) based on person-years incidence to explore the relative importance of different anatomical sites (oropharynx, urethra and anorectum) or different sexual practices. We calculated the relative incidence as the rate of incidence cases by different anatomical sites (oropharynx, urethra and anorectum) or sexual practices (numerators) and the sum of all *M. Genitalium* cases in a year (denominator).

### Uncertainty and sensitivity analysis

Several natural history parameters of *M. genitalium* were uncertain (e.g. duration of infection)^20,31^, and so is the frequency of sexual practices in MSM^17^. To evaluate the stability of our results to uncertainty, we conducted sensitivity analyses on the *M. genitalium* models by varying duration of infection (reduced to half the duration of asymptomatic oropharyngeal and anal infection) and frequency of sexual practices (increased to double or half the days of sexual practices including anal sex and oral sex).

## Supplementary Information

Supplementary Information 1.

Supplementary Information 2.

## Data Availability

All data analysed during this study are included in this article and its additional file.
