# Supplementary material for: Modelling the multiple anatomical site transmission of Mycoplasma genitalium among men who have sex with men in Australia
Source: Sci Rep. 2021 May 27;11:11087. doi: 10.1038/s41598-021-90627-3 (PMC8160207; doi:10.1038/s41598-021-90627-3)
Supplement: Supplementary file 1 — Supplementary Information 1. [file 41598_2021_90627_MOESM1_ESM.docx]

**Modelling the multiple anatomical site transmission of Mycoplasma genitalium among men who have sex with men in Australia**

**Running Title:** The multiple anatomical site transmission of *Mycoplasma genitalium*

**Authors:** Xianglong Xu^1- 3^, Catriona S Bradshaw ^2,3^, Eric P.F. Chow ^2-4^, Jason J. Ong^1- 3^, Jane S Hocking^1,4^, Christopher K. Fairley ^1-3^, Lei Zhang^1-3,5*^

**Affiliations**:

1. China Australia Joint Research Center for Infectious Diseases, School of Public Health, Xi'an Jiaotong University Health Science Centre, Xi'an, Shaanxi, People's Republic of China.
2. Melbourne Sexual Health Centre, Alfred Health, Melbourne, Australia.
3. Central Clinical School, Faculty of Medicine, Nursing and Health Sciences, Monash University, Melbourne, Australia.
4. Centre for Epidemiology and Biostatistics, Melbourne School of Population and Global Health, The University of Melbourne, Melbourne, Australia
5. Department of Epidemiology and Biostatistics, College of Public Health, Zhengzhou University, Zhengzhou, Henan, People's Republic of China.

* Corresponding author

Correspondence to Dr. Lei Zhang, China Australia Joint Research Center for Infectious Diseases, School of Public Health, Xi'an Jiaotong University Health Science Centre, Xi'an, Shaanxi, 710061, People's Republic of China. lei.zhang1@monash.edu (e-mail); +86-29-8265-5135(telephone, and fax numbers).

**Supplementary Results**

**The model-estimated anatomical per-act transmissibility**


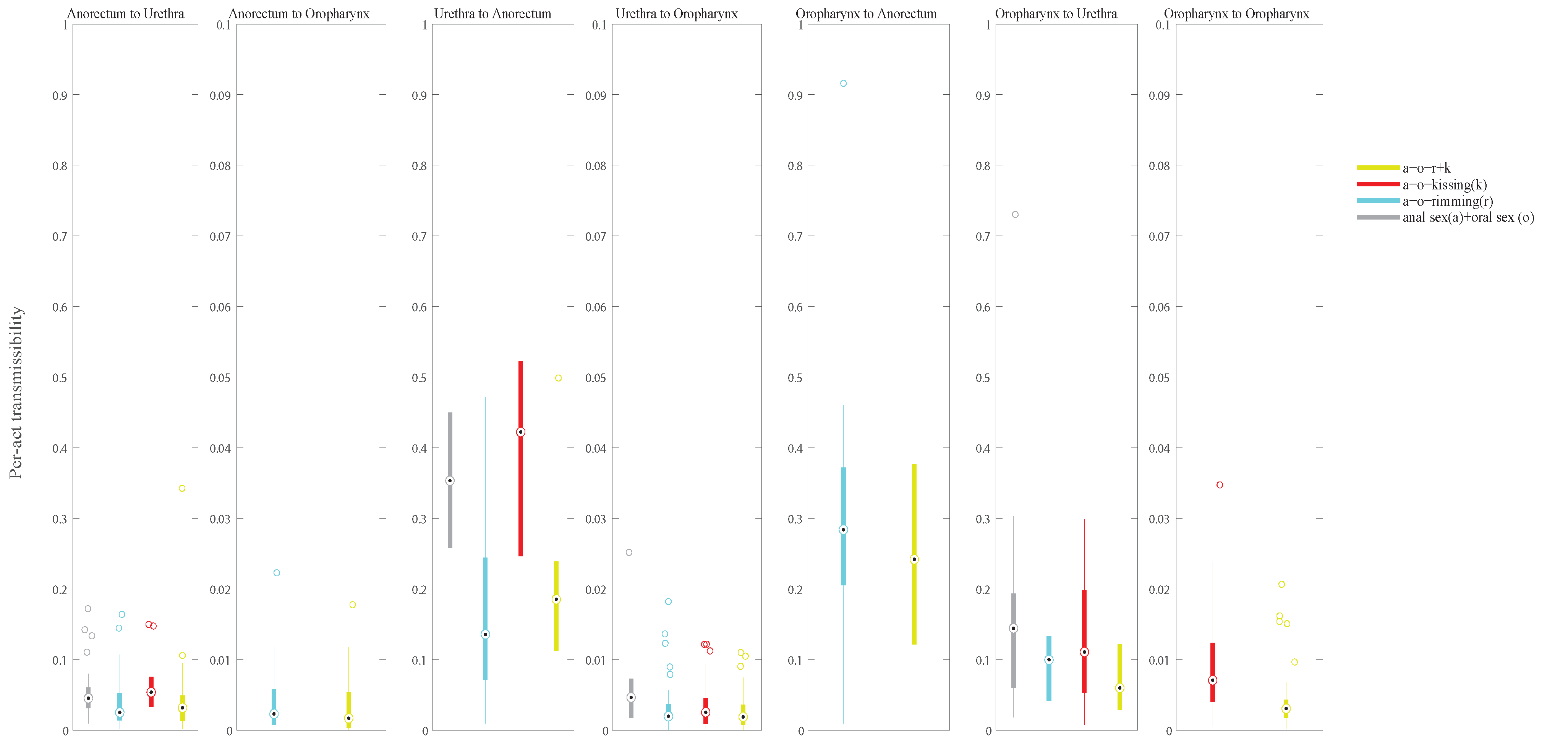


Figure S1. The model-estimated anatomical per-act transmissibility; Model 1: Oral sex and anal sex only; Model 2: Oral sex and anal sex and rimming only; Model3: Oral sex and anal sex and kissing only; Model 4: Oral sex and anal sex and rimming and kissing

**Sensitivity analyses of anal sex and oral sex only model**


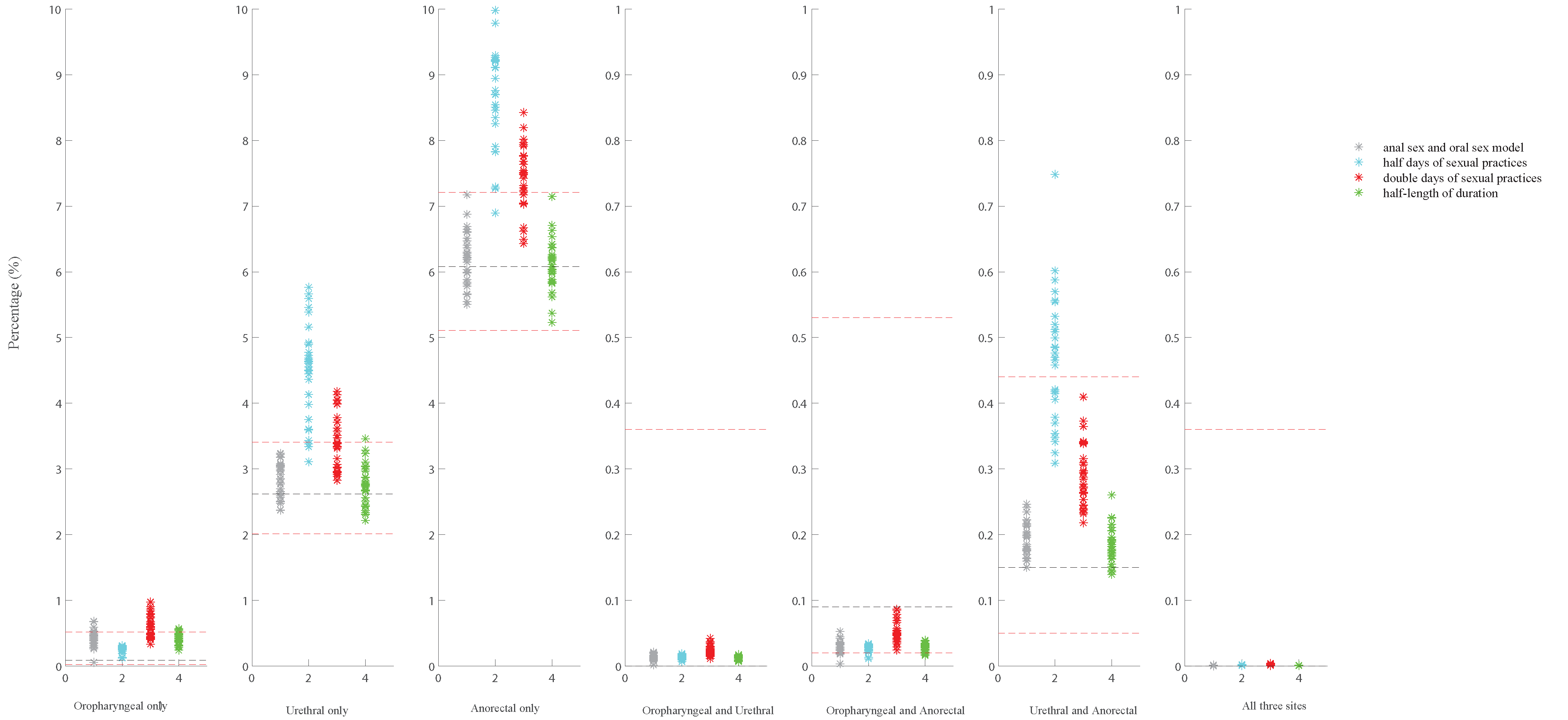


Figure S2. Sensitivity analyses of the estimated percentage of specific anatomical sites positive for *Mycoplasma genitalium* for anal sex and oral sex model and the 95% confidence intervals for the observed site-specific positivity; a. anal sex and oral sex model; b. half days of sexual practices; c. double days of sexual practices; d. half-length of duration


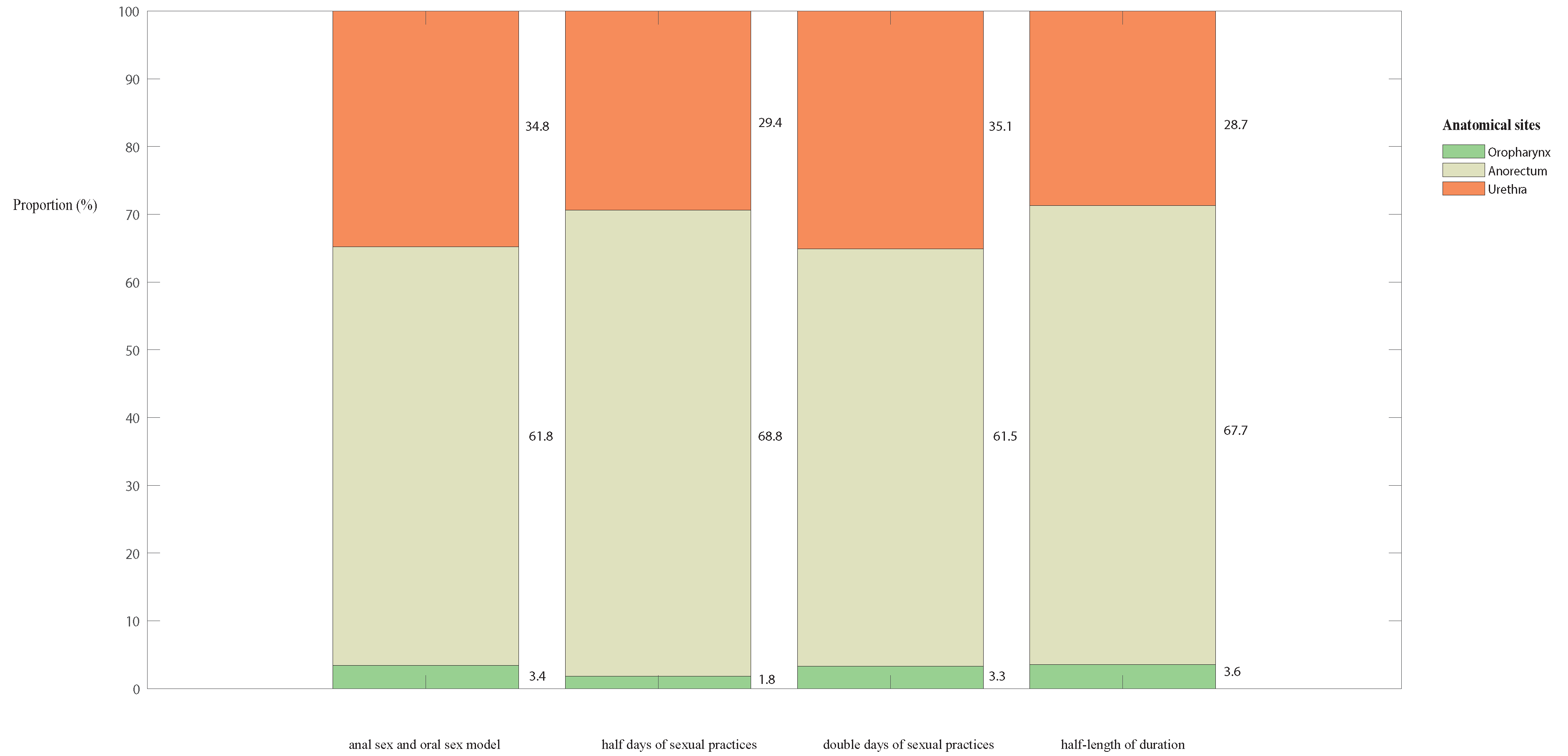


Figure S3a. Sensitivity analysis of the estimated proportion of incidence *Mycoplasma genitalium* cases that occur at the oropharynx, anorectum or urethra in MSM from the anal sex and oral sex model; a. anal sex and oral sex model; b. half days of sexual practices; c. double days of sexual practices; d. half-length of duration


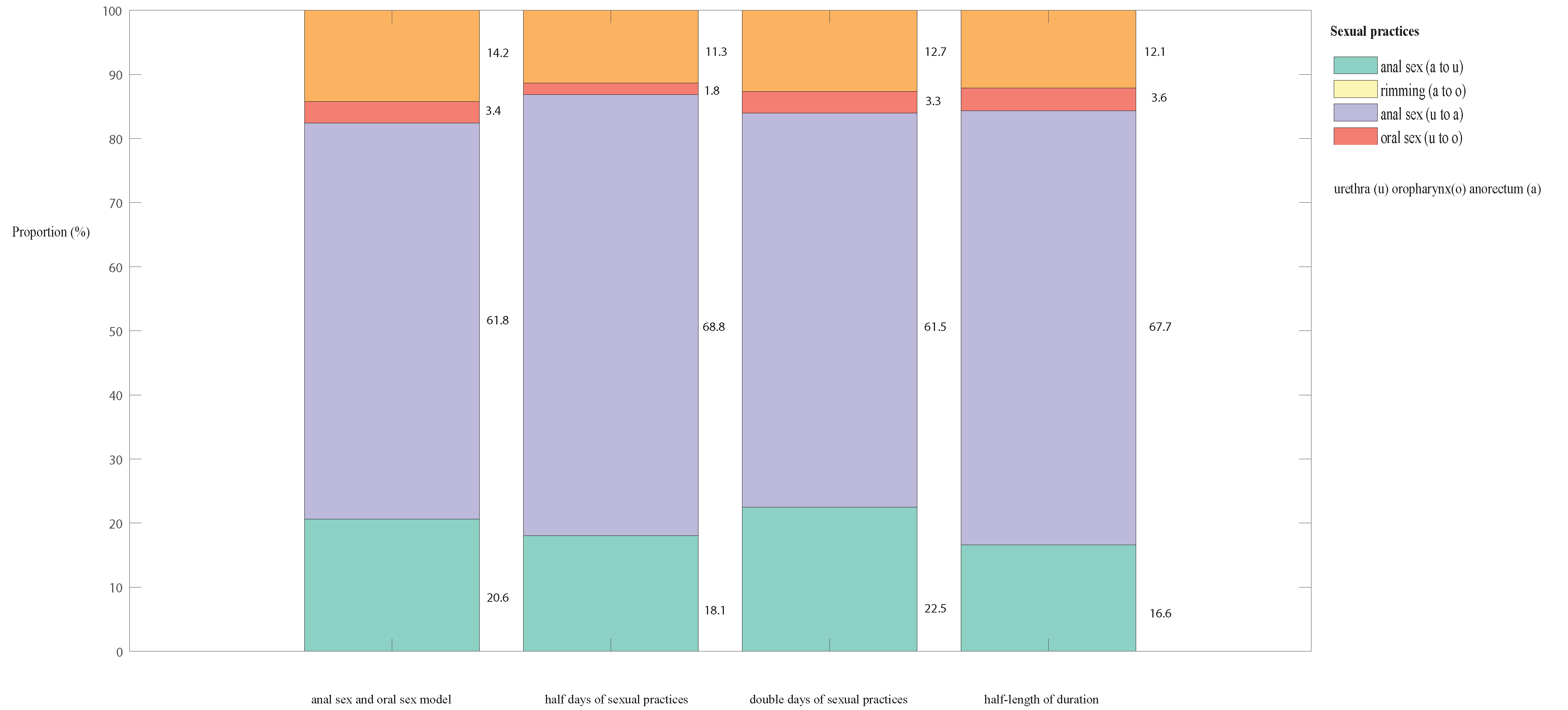


Figure S3b. Sensitivity analysis of the estimated proportion of incidence *Mycoplasma genitalium* cases caused by sexual practices in MSM from the anal sex and oral sex model; a. anal sex and oral sex model; b. half days of sexual practices; c. double days of sexual practices; d. half-length of duration


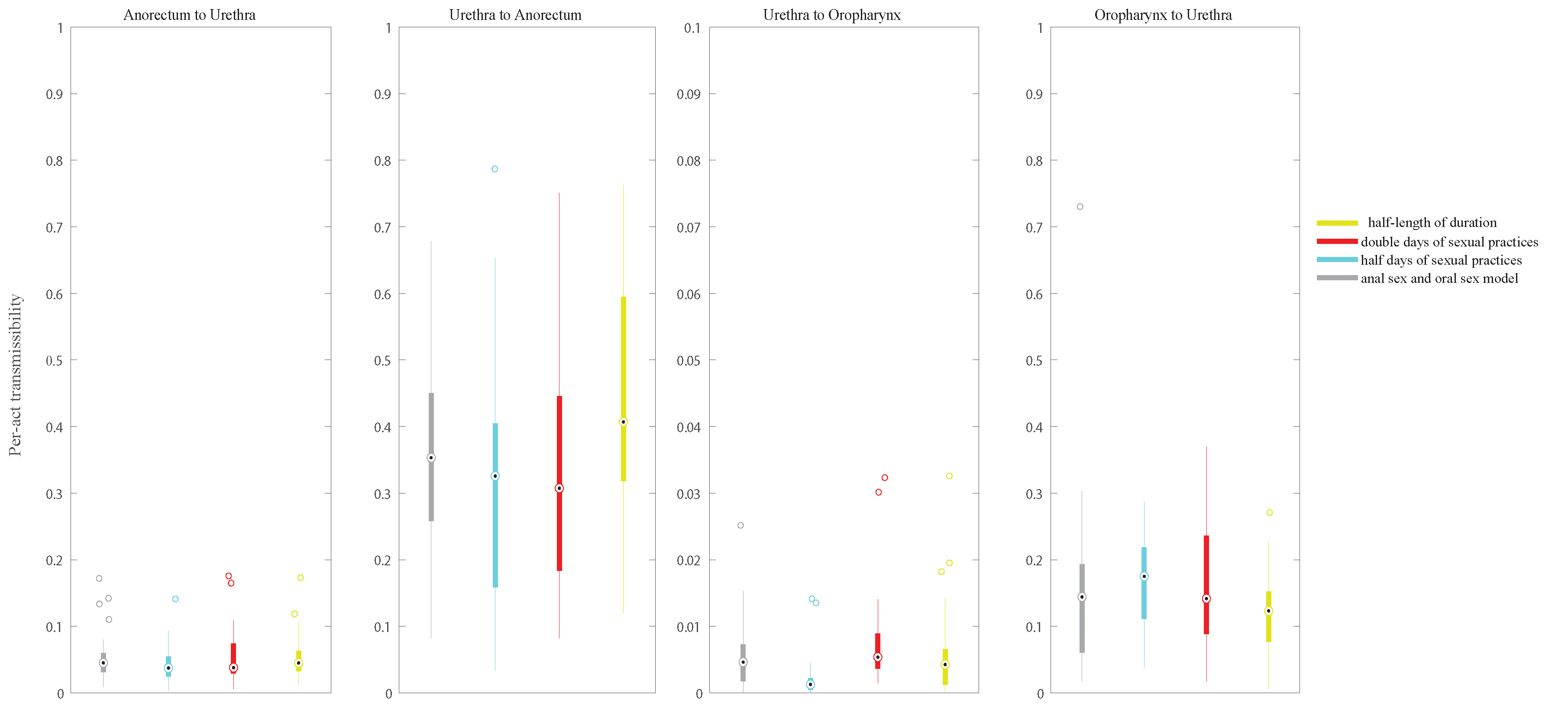


Figure S4. Sensitivity analysis of the estimated anatomical per-act transmissibility of *Mycoplasma genitalium*; a. anal sex and oral sex model; b. half days of sexual practices; c. double days of sexual practices; d. half-length of duration

**Performance of the fitting for empirical *Mycoplasma genitalium* prevalence data**

Table S1. Sum of squared error of four *Mycoplasma genitalium* models

| **Models** | **Sexual practices** | **Sum of Squared Error** **(×10^-6^), *Footnote* a** | **Statistical analysis, *Footnote* c** |
| --- | --- | --- | --- |
|  |  | **Mean, 95% Confidence Interval, *Footnote* b** |  |
| Model 1 | Oral sex and anal sex | 06.43(95%CI 03.38- 7.56) | Reference group |
| Model 2 | Oral sex and anal sex and rimming | 11.51(95%CI 3.57-14.77) | Model 2 vs. Model 1 (p<0.001) |
| Model 3 | Oral sex and anal sex and kissing | 6.08(95%CI 2.17-7.20) | Model 3 vs. Model 1 (p =0.4064) |
| Model 4 | Oral sex and anal sex and rimming and kissing | 10.04(95%CI 1.12-12.79) | Model 4 vs. Model 1 (p<0.001) |

**Footnote:**

1. Sum of squared error measures the total deviation of the calibrated seven prevalence values (single-site infections including only oropharynx infection, only anorectal infection, only urethral infection, and multi-site infections including multi-site infection at oropharyngeal and urethral only, multi-site infection at oropharynx and rectum only, multi-site infection at urethral and rectal only, and multi-site infection at urethral and anorectal and oropharyngeal) from the fit to the observed seven prevalence values.
2. Values close to 0 indicate that the *Mycoplasma genitalium* model has a smaller error.
3. An independent-samples t-test was used to analyze the difference on means of sum of squared error between two *Mycoplasma genitalium* models

**The estimated incidence at oropharynx, urethra and anorectum**

*M. genitalium* incidence in MSM was 6.63 (95% CI 5.14-11.13) per 100 person-years. The model 1 also estimated *M. genitalium* incidence was 4.17(95%CI 2.60- 6.58) per 100 person-years at the anorectum, 1.78(95%CI 1.05- 5.66) per 100 person-years at the urethra, and 0.19 (95%CI 0.03- 0.66) per 100 person-years at the oropharynx. Model 1 also estimated that the proportion of incidence of *M. genitalium* at the oropharynx, anorectum or urethra.

Table S2. The estimated incidence at oropharynx, urethra and anorectum (per 100 person-years)

|  | Oropharynx | Anorectum | Urethra | Overall |
| --- | --- | --- | --- | --- |
| Model 1 | 0.19 (95%CI 0.03- 0.66) | 4.17(95%CI 2.60- 6.58) | 1.78(95%CI 1.05- 5.66) | 6.63(95%CI 5.14-11.13) |
| Model 2 | 0.22(95%CI 0.06-1.21) | 3.82(95%CI 2.23-9.41) | 1.79(95%CI 0.95- 6.64) | 6.54(95%CI 3.93-12.96) |
| Model 3 | 0.20(95%CI 0.08- 0.80) | 4.27(95%CI 2.70- 7.24) | 1.85(95%CI 1.07- 7.07) | 6.94(95%CI 4.54-12.68) |
| Model 4 | 0.24(95%CI 0.04-0.85) | 3.83(95%CI 2.46- 6.75) | 1.71(95%CI0.86-6.01) | 5.83(95%CI 3.59-10.59) |

**Fitted parameters of models**

Table S3. Fitted parameters of model 1: Oral sex and anal sex only

| **Parameters** | **Mean** | **95%CI** | |
| --- | --- | --- | --- |
| %, Consistent condom usage in anal sex in past 12 months | 51.13 | 35.41 | 58.76 |
| %, Condom efficacy in preventing transmission | 87.88 | 81.46 | 94.64 |
| days, Frequency of kissing | 6.89 | 2.28 | 12.80 |
| days, Frequency of oral sex | 12.21 | 3.02 | 26.54 |
| days, Frequency of rimming | 37.05 | 7.32 | 78.18 |
| days, Frequency of anal sex | 25.47 | 6.77 | 47.01 |
| weeks, Infection duration of *Mycoplasma genitalium* at the throat (asymptomatic infection) | 116.08 | 38.57 | 215.53 |
| weeks, Infection duration of *Mycoplasma genitalium* at urethra (symptomatic infection) | 1.48 | 1.05 | 1.97 |
| weeks, Infection duration of *Mycoplasma genitalium* at urethra (asymptomatic infection) | 91.24 | 31.62 | 151.49 |
| %, Proportion of urethral *Mycoplasma genitalium* infections that are asymptomatic | 85.08 | 80.28 | 89.90 |
| %, Proportion of anal *Mycoplasma genitalium* infections that are asymptomatic | 75.52 | 61.55 | 93.38 |
| weeks, Infection duration at anus (symptomatic infection) | 1.57 | 1.12 | 1.91 |
| weeks, Infection duration of *Mycoplasma genitalium* at the anus (asymptomatic infection) | 103.60 | 57.35 | 127.86 |
| %, Infection prevalence of *Mycoplasma genitalium* only at Oropharynx | 0.43 | 0.11 | 0.68 |
| %, Infection prevalence of *Mycoplasma genitalium* only at Urethral | 2.85 | 2.37 | 3.23 |
| %, Infection prevalence of *Mycoplasma genitalium* only at Rectum | 6.24 | 5.52 | 7.10 |
| %, Infection prevalence of *Mycoplasma genitalium* at Oropharynx & Urethra | 0.01 | 0.00 | 0.02 |
| %, Infection prevalence of *Mycoplasma genitalium* at Oropharynx & Rectum | 0.03 | 0.01 | 0.05 |
| %, Infection prevalence of *Mycoplasma genitalium* at Urethra & Rectum | 0.20 | 0.15 | 0.25 |
| %, Infection prevalence of *Mycoplasma genitalium* at Oropharynx & Urethra & Rectum | 0.00 | 0.00 | 0.00 |

Table S4. Fitted parameters of mode 2: Oral sex and anal sex and rimming only

| **Parameters** | **Mean** | **95%CI** | |
| --- | --- | --- | --- |
| %, Consistent condom usage in anal sex in past 12 months | 48.42 | 35.78 | 58.23 |
| %, Condom efficacy in preventing transmission | 87.81 | 81.19 | 94.18 |
| days, Frequency of kissing | 7.84 | 0.72 | 13.01 |
| days, Frequency of oral sex | 9.32 | 1.06 | 25.45 |
| days, Frequency of rimming | 26.38 | 1.91 | 70.00 |
| days, Frequency of anal sex | 15.24 | 1.16 | 48.47 |
| weeks, Infection duration of *Mycoplasma genitalium* at the throat (asymptomatic infection) | 121.98 | 32.35 | 230.79 |
| weeks, Infection duration of *Mycoplasma genitalium* at urethra (symptomatic infection) | 1.41 | 1.03 | 1.97 |
| weeks, Infection duration of *Mycoplasma genitalium* at urethra (asymptomatic infection) | 93.31 | 24.27 | 151.49 |
| %, Proportion of urethral *Mycoplasma genitalium* infections that are asymptomatic | 85.18 | 80.58 | 88.94 |
| %, Proportion of anal *Mycoplasma genitalium* infections that are asymptomatic | 79.78 | 62.03 | 95.42 |
| weeks, Infection duration at anus (symptomatic infection) | 1.47 | 1.04 | 1.96 |
| weeks, Infection duration of *Mycoplasma genitalium* at the anus (asymptomatic infection) | 103.74 | 54.89 | 130.88 |
| %, Infection prevalence of *Mycoplasma genitalium* only at Oropharynx | 0.54 | 0.18 | 0.70 |
| %, Infection prevalence of *Mycoplasma genitalium* only at Urethral | 2.66 | 2.24 | 3.15 |
| %, Infection prevalence of *Mycoplasma genitalium* only at Rectum | 6.13 | 5.28 | 6.87 |
| %, Infection prevalence of *Mycoplasma genitalium* at Oropharynx & Urethra | 0.02 | 0.01 | 0.02 |
| %, Infection prevalence of *Mycoplasma genitalium* at Oropharynx & Rectum | 0.04 | 0.01 | 0.05 |
| %, Infection prevalence of *Mycoplasma genitalium* at Urethra & Rectum | 0.18 | 0.14 | 0.23 |
| %, Infection prevalence of *Mycoplasma genitalium* at Oropharynx & Urethra & Rectum | 0.00 | 0.00 | 0.00 |

Table S5. Fitted parameters of mode3: Oral sex and anal sex and kissing only

| **Parameters** | **Mean/95%CI** | | |
| --- | --- | --- | --- |
| %, Consistent condom usage in anal sex in past 12 months | 45.39 | 35.51 | 58.71 |
| %, Condom efficacy in preventing transmission | 88.60 | 81.58 | 94.30 |
| days, Frequency of kissing | 7.27 | 0.62 | 12.07 |
| days, Frequency of oral sex | 9.28 | 0.93 | 26.67 |
| days, Frequency of rimming | 38.21 | 1.61 | 71.98 |
| days, Frequency of anal sex | 27.17 | 3.87 | 53.61 |
| weeks, Infection duration of *Mycoplasma genitalium* at the throat (asymptomatic infection) | 99.50 | 29.95 | 226.85 |
| weeks, Infection duration of *Mycoplasma genitalium* at urethra (symptomatic infection) | 1.50 | 1.03 | 1.90 |
| weeks, Infection duration of *Mycoplasma genitalium* at urethra (asymptomatic infection) | 89.65 | 23.18 | 150.55 |
| %, Proportion of urethral *Mycoplasma genitalium* infections that are asymptomatic | 84.75 | 80.80 | 89.78 |
| %, Proportion of anal *Mycoplasma genitalium* infections that are asymptomatic | 84.79 | 62.56 | 96.16 |
| weeks, Infection duration at anus (symptomatic infection) | 1.48 | 1.06 | 1.93 |
| weeks, Infection duration of *Mycoplasma genitalium* at the anus (asymptomatic infection) | 92.41 | 64.45 | 125.47 |
| %, Infection prevalence of *Mycoplasma genitalium* only at Oropharynx | 0.46 | 0.29 | 0.60 |
| %, Infection prevalence of *Mycoplasma genitalium* only at Urethral | 2.73 | 2.26 | 3.33 |
| %, Infection prevalence of *Mycoplasma genitalium* only at Rectum | 6.04 | 5.43 | 6.80 |
| %, Infection prevalence of *Mycoplasma genitalium* at Oropharynx & Urethra | 0.01 | 0.01 | 0.02 |
| %, Infection prevalence of *Mycoplasma genitalium* at Oropharynx & Rectum | 0.03 | 0.02 | 0.04 |
| %, Infection prevalence of *Mycoplasma genitalium* at Urethra & Rectum | 0.18 | 0.16 | 0.24 |
| %, Infection prevalence of *Mycoplasma genitalium* at Oropharynx & Urethra & Rectum | 0.00 | 0.00 | 0.00 |

Table S6. Fitted parameters of mode 4: Oral sex and anal sex and rimming and kissing

| **Parameters** | **Mean/95%CI** | | |
| --- | --- | --- | --- |
| %, Consistent condom usage in anal sex in past 12 months | 45.18 | 35.65 | 57.05 |
| %, Condom efficacy in preventing transmission | 89.20 | 80.59 | 94.44 |
| days, Frequency of kissing | 5.87 | 0.35 | 11.17 |
| days, Frequency of oral sex | 9.87 | 0.58 | 26.86 |
| days, Frequency of rimming | 21.49 | 1.37 | 77.35 |
| days, Frequency of anal sex | 17.56 | 2.36 | 50.85 |
| weeks, Infection duration of *Mycoplasma genitalium* at the throat (asymptomatic infection) | 128.80 | 40.87 | 236.33 |
| weeks, Infection duration of *Mycoplasma genitalium* at urethra (symptomatic infection) | 1.51 | 1.01 | 1.98 |
| weeks, Infection duration of *Mycoplasma genitalium* at urethra (asymptomatic infection) | 101.33 | 28.02 | 154.68 |
| %, Proportion of urethral *Mycoplasma genitalium* infections that are asymptomatic | 85.42 | 81.59 | 88.81 |
| %, Proportion of anal *Mycoplasma genitalium* infections that are asymptomatic | 83.60 | 63.85 | 95.93 |
| weeks, Infection duration at anus (symptomatic infection) | 1.48 | 1.05 | 1.92 |
| weeks, Infection duration of *Mycoplasma genitalium* at the anus (asymptomatic infection) | 99.83 | 61.29 | 131.05 |
| %, Infection prevalence of *Mycoplasma genitalium* only at Oropharynx | 0.62 | 0.19 | 0.74 |
| %, Infection prevalence of *Mycoplasma genitalium* only at Urethral | 2.64 | 2.14 | 3.34 |
| %, Infection prevalence of *Mycoplasma genitalium* only at Rectum | 6.10 | 5.30 | 6.89 |
| %, Infection prevalence of *Mycoplasma genitalium* at Oropharynx & Urethra | 0.02 | 0.01 | 0.02 |
| %, Infection prevalence of *Mycoplasma genitalium* at Oropharynx & Rectum | 0.04 | 0.01 | 0.05 |
| %, Infection prevalence of *Mycoplasma genitalium* at Urethra & Rectum | 0.17 | 0.13 | 0.25 |
| %, Infection prevalence of *Mycoplasma genitalium* at Oropharynx & Urethra & Rectum | 0.00 | 0.00 | 0.00 |

Table S7. Fitted parameters of mode 1: half days of sexual practices

| **Parameters** | **Mean/95%CI** | | |
| --- | --- | --- | --- |
| %, Consistent condom usage in anal sex in past 12 months | 45.67 | 35.78 | 58.13 |
| %, Condom efficacy in preventing transmission | 87.79 | 81.45 | 94.52 |
| days, Frequency of kissing | 6.28 | 0.22 | 12.48 |
| days, Frequency of oral sex | 8.95 | 1.08 | 26.12 |
| days, Frequency of rimming | 44.09 | 1.85 | 75.67 |
| days, Frequency of anal sex | 25.50 | 3.13 | 50.63 |
| weeks, Infection duration of *Mycoplasma genitalium* at the throat (asymptomatic infection) | 133.67 | 28.29 | 226.47 |
| weeks, Infection duration of *Mycoplasma genitalium* at urethra (symptomatic infection) | 1.43 | 1.04 | 1.95 |
| weeks, Infection duration of *Mycoplasma genitalium* at urethra (asymptomatic infection) | 107.29 | 69.43 | 152.40 |
| %, Proportion of urethral *Mycoplasma genitalium* infections that are asymptomatic | 85.57 | 80.92 | 89.68 |
| %, Proportion of anal *Mycoplasma genitalium* infections that are asymptomatic | 82.03 | 62.50 | 96.43 |
| weeks, Infection duration at anus (symptomatic infection) | 1.41 | 1.06 | 1.97 |
| weeks, Infection duration of *Mycoplasma genitalium* at the anus (asymptomatic infection) | 96.21 | 61.81 | 126.65 |
| %, Infection prevalence of *Mycoplasma genitalium* only at Oropharynx | 0.00 | 0.00 | 0.00 |
| %, Infection prevalence of *Mycoplasma genitalium* only at Urethral | 5.07 | 3.32 | 6.40 |
| %, Infection prevalence of *Mycoplasma genitalium* only at Rectum | 9.24 | 6.59 | 11.17 |
| %, Infection prevalence of *Mycoplasma genitalium* at Oropharynx & Urethra | 0.00 | 0.00 | 0.00 |
| %, Infection prevalence of *Mycoplasma genitalium* at Oropharynx & Rectum | 0.00 | 0.00 | 0.00 |
| %, Infection prevalence of *Mycoplasma genitalium* at Urethra & Rectum | 0.77 | 0.20 | 1.25 |
| %, Infection prevalence of *Mycoplasma genitalium* at Oropharynx & Urethra & Rectum | 0.00 | 0.00 | 0.00 |

Table S8. Fitted parameters of mode 1: double days of sexual practices

| **Parameters** | **Mean/95%CI** | | |
| --- | --- | --- | --- |
| %, Consistent condom usage in anal sex in past 12 months | 47.47 | 36.28 | 56.22 |
| %, Condom efficacy in preventing transmission | 87.45 | 80.56 | 93.48 |
| days, Frequency of kissing | 4.76 | 0.94 | 11.67 |
| days, Frequency of oral sex | 14.74 | 4.05 | 27.54 |
| days, Frequency of rimming | 36.18 | 3.51 | 69.01 |
| days, Frequency of anal sex | 21.18 | 4.45 | 51.36 |
| weeks, Infection duration of *Mycoplasma genitalium* at the throat (asymptomatic infection) | 115.47 | 45.39 | 209.89 |
| weeks, Infection duration of *Mycoplasma genitalium* at urethra (symptomatic infection) | 1.44 | 1.04 | 1.89 |
| weeks, Infection duration of *Mycoplasma genitalium* at urethra (asymptomatic infection) | 80.74 | 22.67 | 153.64 |
| %, Proportion of urethral *Mycoplasma genitalium* infections that are asymptomatic | 83.78 | 80.88 | 89.23 |
| %, Proportion of anal *Mycoplasma genitalium* infections that are asymptomatic | 77.33 | 62.20 | 95.72 |
| weeks, Infection duration at anus (symptomatic infection) | 1.58 | 1.07 | 1.98 |
| weeks, Infection duration of *Mycoplasma genitalium* at the anus (asymptomatic infection) | 89.16 | 51.02 | 120.97 |
| %, Infection prevalence of *Mycoplasma genitalium* only at Oropharynx | 0.47 | 0.13 | 0.89 |
| %, Infection prevalence of *Mycoplasma genitalium* only at Urethral | 3.47 | 2.67 | 4.18 |
| %, Infection prevalence of *Mycoplasma genitalium* only at Rectum | 7.40 | 6.55 | 8.72 |
| %, Infection prevalence of *Mycoplasma genitalium* at Oropharynx & Urethra | 0.34 | 0.07 | 0.66 |
| %, Infection prevalence of *Mycoplasma genitalium* at Oropharynx & Rectum | 0.64 | 0.15 | 0.97 |
| %, Infection prevalence of *Mycoplasma genitalium* at Urethra & Rectum | 0.37 | 0.10 | 0.56 |
| %, Infection prevalence of *Mycoplasma genitalium* at Oropharynx & Urethra & Rectum | 0.35 | 0.04 | 0.66 |

Table S9. Fitted parameters of mode 1: half-length of duration

| **Parameters** | **Mean/95%CI** | | |
| --- | --- | --- | --- |
| %, Consistent condom usage in anal sex in past 12 months | 50.50 | 39.34 | 56.37 |
| %, Condom efficacy in preventing transmission | 87.84 | 80.29 | 93.87 |
| days, Frequency of kissing | 6.69 | 0.25 | 12.27 |
| days, Frequency of oral sex | 12.92 | 1.07 | 26.38 |
| days, Frequency of rimming | 46.76 | 3.93 | 68.70 |
| days, Frequency of anal sex | 31.96 | 5.60 | 52.59 |
| weeks, Infection duration of *Mycoplasma genitalium* at the throat (asymptomatic infection) | 148.82 | 27.43 | 231.23 |
| weeks, Infection duration of *Mycoplasma genitalium* at urethra (symptomatic infection) | 1.54 | 1.06 | 1.93 |
| weeks, Infection duration of *Mycoplasma genitalium* at urethra (asymptomatic infection) | 97.30 | 38.51 | 149.37 |
| %, Proportion of urethral *Mycoplasma genitalium* infections that are asymptomatic | 86.02 | 80.86 | 89.06 |
| %, Proportion of anal *Mycoplasma genitalium* infections that are asymptomatic | 79.03 | 60.78 | 91.53 |
| weeks, Infection duration at anus (symptomatic infection) | 1.37 | 1.07 | 1.93 |
| weeks, Infection duration of *Mycoplasma genitalium* at the anus (asymptomatic infection) | 91.26 | 59.84 | 129.20 |
| %, Infection prevalence of *Mycoplasma genitalium* only at Oropharynx | 0.23 | 0.04 | 0.48 |
| %, Infection prevalence of *Mycoplasma genitalium* only at Urethral | 2.61 | 2.08 | 3.34 |
| %, Infection prevalence of *Mycoplasma genitalium* only at Rectum | 6.46 | 5.26 | 7.08 |
| %, Infection prevalence of *Mycoplasma genitalium* at Oropharynx & Urethra | 0.22 | 0.04 | 0.34 |
| %, Infection prevalence of *Mycoplasma genitalium* at Oropharynx & Rectum | 0.28 | 0.06 | 0.43 |
| %, Infection prevalence of *Mycoplasma genitalium* at Urethra & Rectum | 0.23 | 0.08 | 0.43 |
| %, Infection prevalence of *Mycoplasma genitalium* at Oropharynx & Urethra & Rectum | 0.18 | 0.01 | 0.30 |
