# Supplementary material for: Modelling the multiple anatomical site transmission of Mycoplasma genitalium among men who have sex with men in Australia
Source: Sci Rep. 2021 May 27;11:11087. doi: 10.1038/s41598-021-90627-3 (PMC8160207; doi:10.1038/s41598-021-90627-3)
Supplement: Supplementary file 2 — Supplementary Information 2. [file 41598_2021_90627_MOESM2_ESM.docx]

**Modelling the multiple anatomical site transmission of Mycoplasma genitalium among men who have sex with men in Australia**

**Running Title:** The multiple anatomical site transmission of *Mycoplasma genitalium*

**Authors:** Xianglong Xu^1- 3^, Catriona S Bradshaw ^2,3^, Eric P.F. Chow ^2-4^, Jason J. Ong^1- 3^, Jane S Hocking^1,4^, Christopher K. Fairley ^1-3^, Lei Zhang^1-3,5*^

**Affiliations**:

1. China Australia Joint Research Center for Infectious Diseases, School of Public Health, Xi'an Jiaotong University Health Science Centre, Xi'an, Shaanxi, People's Republic of China.
2. Melbourne Sexual Health Centre, Alfred Health, Melbourne, Australia.
3. Central Clinical School, Faculty of Medicine, Nursing and Health Sciences, Monash University, Melbourne, Australia.
4. Centre for Epidemiology and Biostatistics, Melbourne School of Population and Global Health, The University of Melbourne, Melbourne, Australia
5. Department of Epidemiology and Biostatistics, College of Public Health, Zhengzhou University, Zhengzhou, Henan, People's Republic of China.

* Corresponding author

Correspondence to Dr. Lei Zhang, China Australia Joint Research Center for Infectious Diseases, School of Public Health, Xi'an Jiaotong University Health Science Centre, Xi'an, Shaanxi, 710061, People's Republic of China. lei.zhang1@monash.edu (e-mail); +86-29-8265-5135(telephone, and fax numbers).

**Ordinary differential equations**

Table S1. *Mycoplasma genitalium* transmission model symbols and description

| **Symbol** | **Description** |
| --- | --- |
| I | Infected MSM |
| I_o_ | Number of infected at oropharynx |
| I_u_ | Number of infected at the urethra |
| I_a_ | Number of infected at the anorectum |
| I_ou_ | Number of infected at the oropharynx and urethra |
| I_ua_ | Number of infected at the anorectum, and urethra |
| I_oa_ | Number of infected at oropharynx and anorectum |
| I_oua_ | Number of infected at oropharynx, anorectum, and urethra |
| P | Prevalence |
| P_o_ | The prevalence of infected only at the oropharynx |
| P_a_ | The prevalence of infected only at the anorectum |
| P_u_ | The prevalence of infected only at the urethra |
| P_ou_ | The prevalence of infected only at oropharynx and urethra |
| P_ua_ | The prevalence of infected only at the anorectum, and urethra |
| P_oa_ | The prevalence of infected only at the oropharynx and anorectum |
| P_oua_ | The prevalence of infected at oropharynx, anorectum, and urethra |
| $\beta$ | Per-act transmission probability |
| λ | The rate of conversion from susceptible to infected individuals, it is a function of per-act transmission probability, frequency of sex acts, condom use and condom efficacy, the product of λ and site-specific prevalence defines the ‘force of infection’ at the specific site |
| λ_oo_ | kissing (oropharynx to oropharynx) |
| λ_ao_ | rimming (anorectum to oropharynx) |
| λ_oa_ | rimming (oropharynx to anorectum) |
| λ_au_ | anal sex (anorectum to urethra) |
| λ_ua_ | anal sex (urethra to anorectum) |
| λ_uo_ | oral sex (urethra to oropharynx) |
| λ_ou_ | oral sex (oropharynx to urethra) |
| γ | The rate of infection clearance |
| $\gamma_{u}$ | The rate of oropharyngeal infection clearance |
| $\gamma_{a}$ | The rate of anorectal infection clearance |
| $\gamma_{o}$ | The rate of urethral infection clearance |
| C | Consistent condom usage in anal sex |
| $\varepsilon_{c}$ | The efficacy of condom in preventing transmission of infection |
| f | The frequency of sexual practices (kissing, rimming, oral sex and anal sex) |
| incid.au | New infection caused by anal sex (anorectum to urethra) |
| incid.ao | New infection caused by rimming (anorectum to oropharynx) |
| incid.ua | New infection caused by anal sex (urethra to anorectum) |
| incid.uo | New infection caused by oral sex (urethra to oropharynx) |
| incid.oa | New infection caused by rimming (oropharynx to anorectum) |
| incid.ou | New infection caused by oral sex (oropharynx to urethra) |
| incid.oo | New infection caused by kissing (oropharynx to oropharynx) |
| incid.o | New infection occurs at the oropharynx |
| incid.a | New infection occurs at the anorectum |
| incid.u | New infection occurs at the urethra |

The ordinary differential equations as follow:

$${\lambda_{ij}=1-\left( 1-\beta_{ij}\cdot(1-\varepsilon_{c}\cdot C) \right)}^{\frac{f_{ij}}{2}})$$

$$N=S+I_{o}+I_{u}+I_{a}+I_{ou}+I_{ua}+I_{oa}+I_{oua}$$

$$P_{o}=I_{o}/N$$

$$P_{u}=I_{u}/N$$

$$P_{a}=I_{a}/N$$

$$P_{ou}=I_{ou}/N$$

$$P_{ua}=I_{ua}/N$$

$$P_{oa}=I_{oa}/N$$

$$P_{oua}=I_{oua}/N$$

$$P_{o\_all}=P_{o}+P_{ou}+P_{oa}+P_{oua}$$

$$P_{a\_all}=P_{a}+P_{oa}+P_{ua}+P_{oua}$$

$$P_{u\_all}=P_{u}+P_{ou}+P_{ua}+P_{oua}$$

$$P_{ou\_all}=P_{ou}+P_{oua}$$

$$P_{ua\_all}=P_{ua}+P_{oua}$$

$$P_{oa\_all}=P_{oa}+P_{oua}$$

$$\frac{dP_{s}}{dt}=-a1\cdot P_{s}+\gamma_{o}\cdot P_{o}-a2\cdot P_{s}+\gamma_{a}\cdot P_{a}-a3\cdot P_{s}+\gamma_{u}\cdot P_{u}$$

$$\frac{dP_{o}}{dt}=a1\cdot P_{s}-\gamma_{o}\cdot P_{o}-a2\cdot P_{o}+\gamma_{a}\cdot P_{oa}-a3\cdot P_{o}+\gamma_{u}\cdot P_{ou}$$

$$\frac{dP_{u}}{dt}=a3\cdot P_{s}-\gamma_{u}\cdot P_{u}-a1\cdot P_{u}+\gamma_{o}\cdot P_{ou}-a2\cdot P_{u}+\gamma_{a}\cdot P_{ua}$$

$$\frac{dP_{a}}{dt}=a2\cdot P_{s}-\gamma_{a}\cdot P_{a}-a1\cdot P_{a}+\gamma_{o}\cdot P_{oa}-a3\cdot P_{a}+\gamma_{u}\cdot P_{ua}$$

$$\frac{dP_{ou}}{dt}=a1\cdot P_{u}-\gamma_{o}\cdot P_{ou}+a3\cdot P_{o}-\gamma_{u}\cdot P_{ou}-a2\cdot P_{ou}+\gamma_{a}\cdot P_{oua}$$

$$\frac{dP_{oa}}{dt}= a2\cdot P_{o}-\gamma_{a}\cdot P_{oa}+a1\cdot P_{a}-\gamma_{o}\cdot P_{oa}-a3\cdot P_{oa}+\gamma_{u}\cdot P_{oua}$$

$$\frac{dP_{ua}}{dt}=a2\cdot P_{u}-\gamma_{a}\cdot P_{ua}+a3\cdot P_{a}-\gamma_{u}\cdot P_{ua}-a1\cdot P_{ua}+\gamma_{o}\cdot P_{oua}$$

$$\frac{dP_{oua}}{dt}=a2\cdot P_{ou}-\gamma_{a}\cdot P_{oua}+a1\cdot P_{ua}-\gamma_{o}\cdot P_{oua}+a3\cdot P_{oa}-\gamma_{u}\cdot P_{oua}$$

**Model 1:** **Oral sex and anal sex**

$$a1=\lambda_{uo}{\boldsymbol{\cdot}P}_{u\_all}$$

$$a2=\lambda_{ua}{\boldsymbol{\cdot}P}_{u\_all}$$

$$a3=\lambda_{ou}{\boldsymbol{\cdot}P}_{o\_all}+\lambda_{au}\boldsymbol{\cdot}P_{a\_all}$$

**Estimating *Mycoplasma genitalium* incidence attributed to oral sex and anal sex**

$$incid.au=\lambda_{au}\cdot P_{a\_all}\cdot(Ps+ Po+ Pa+ Poa)$$

$$incid.ua=\lambda_{ua \cdot}P_{u\_all}\cdot(Ps+ Po+ Pu+ Pou)$$

$$incid.uo=\lambda_{uo \cdot}P_{u\_all}\cdot(Ps+ Pu+ Pa+ Pua)$$

$$incid.ou=\lambda_{ou}{\cdot P}_{o\_all}\cdot(Ps+ Po+ Pa+ Poa)$$

***Mycoplasma genitalium* incidence attributed to** **oropharyngeal, anorectal, and urethral infection**

$$incid.o= incid.uo$$

$$incid.a= incid.ua$$

$$incid.u= incid.au+ incid.ou$$

**Model 2: Oral sex and anal sex and rimming**

$$a1=\lambda_{ao}\boldsymbol{\cdot}P_{a\_all}+\lambda_{uo}{\boldsymbol{\cdot}P}_{u\_all}$$

$$a2=\lambda_{oa}\boldsymbol{\cdot}P_{o\_all}+\lambda_{ua}{\boldsymbol{\cdot}P}_{u\_all}$$

$$a3=\lambda_{ou}{\boldsymbol{\cdot}P}_{o\_all}+\lambda_{au}{\boldsymbol{\cdot}P}_{a\_all}$$

**Estimating *Mycoplasma genitalium* incidence attributed to oral sex, anal sex and rimming**

$$incid.au=\lambda_{au}\cdot P_{a\_all}\cdot(Ps+ Po+ Pa+ Poa)$$

$$incid.ao=\lambda_{ao\cdot}P_{a\_all}\cdot(Ps+ Pu+ Pa+ Pua)$$

$$incid.ua=\lambda_{ua \cdot}P_{u\_all}\cdot(Ps+ Po+ Pu+ Pou)$$

$$incid.uo=\lambda_{uo \cdot}P_{u\_all}\cdot(Ps+ Pu+ Pa+ Pua)$$

$$incid.oa=\lambda_{oa \cdot}P_{o\_all}\cdot(Ps+ Po+ Pu+ Pou)$$

$$incid.ou=\lambda_{ou}{\cdot P}_{o\_all}\cdot(Ps+ Po+ Pa+ Poa)$$

***Mycoplasma genitalium* incidence attributed to** **oropharyngeal, anorectal, and urethral infection**

$$incid.o= incid.ao+ incid.uo$$

$$incid.a= incid.ua+ incid.oa$$

$$incid.u= incid.au+ incid.ou$$

**Model 3: Oral sex and anal sex and kissing**

$${a1=\lambda}_{oo}\boldsymbol{\cdot}P_{o\_all}+\lambda_{uo}{\boldsymbol{\cdot}P}_{u\_all}$$

$$a2=\lambda_{ua}{\boldsymbol{\cdot}P}_{u\_all}$$

$$a3=\lambda_{ou}{\boldsymbol{\cdot}P}_{o\_all}+\lambda_{au}{\boldsymbol{\cdot}P}_{a\_all}$$

**Estimating *Mycoplasma genitalium* incidence attributed to oral sex, anal sex and kissing**

$$incid.au=\lambda_{au}\cdot P_{a\_all}\cdot(Ps+ Po+ Pa+ Poa)$$

$$incid.ua=\lambda_{ua \cdot}P_{u\_all}\cdot(Ps+ Po+ Pu+ Pou)$$

$$incid.uo=\lambda_{uo \cdot}P_{u\_all}\cdot(Ps+ Pu+ Pa+ Pua)$$

$$incid.ou=\lambda_{ou}{\cdot P}_{o\_all}\cdot(Ps+ Po+ Pa+ Poa)$$

$$incid.oo=\lambda_{oo}{\cdot P}_{o\_all}\cdot(Ps+ Pu+ Pa+ Pua)$$

***Mycoplasma genitalium* incidence attributed to** **oropharyngeal, anorectal, and urethral infection**

$$incid.o= incid.uo+ incid.oo$$

$$incid.a= incid.ua$$

$$incid.u= incid.au+ incid.ou$$

**Model 4: Oral sex and anal sex and rimming and kissing**

$${a1=\lambda}_{oo}\boldsymbol{\cdot}P_{o\_all}+\lambda_{ao}{\boldsymbol{\cdot}P}_{a\_all}+\lambda_{uo}{\boldsymbol{\cdot}P}_{u\_all}$$

$$a2=\lambda_{oa}{\boldsymbol{\cdot}P}_{o\_all}+\lambda_{ua}{\boldsymbol{\cdot}P}_{u\_all}$$

$$a3=\lambda_{ou}{\boldsymbol{\cdot}P}_{o\_all}+\lambda_{au}{\boldsymbol{\cdot}P}_{a\_all}$$

**Estimating *Mycoplasma genitalium* incidence attributed to oral sex, anal sex, rimming and kissing**

$$incid.au=\lambda_{au}\cdot P_{a\_all}\cdot(Ps+ Po+ Pa+ Poa)$$

$$incid.ao=\lambda_{ao\cdot}P_{a\_all}\cdot(Ps+ Pu+ Pa+ Pua)$$

$$incid.ua=\lambda_{ua \cdot}P_{u\_all}\cdot(Ps+ Po+ Pu+ Pou)$$

$$incid.uo=\lambda_{uo \cdot}P_{u\_all}\cdot(Ps+ Pu+ Pa+ Pua)$$

$$incid.oa=\lambda_{oa \cdot}P_{o\_all}\cdot(Ps+ Po+ Pu+ Pou)$$

$$incid.ou=\lambda_{ou}{\cdot P}_{o\_all}\cdot(Ps+ Po+ Pa+ Poa)$$

$$incid.oo=\lambda_{oo}{\cdot P}_{o\_all}\cdot(Ps+ Pu+ Pa+ Pua)$$

***Mycoplasma genitalium* incidence attributed to** **oropharyngeal, anorectal, and urethral infection**

$$incid.o= incid.ao+ incid.uo+ incid.oo$$

$$incid.a= incid.ua+ incid.oa$$

$$incid.u= incid.au+ incid.ou$$

**Model parameters**

Natural history parameters of *M. genitalium* were more uncertain ^1^; therefore, we used chlamydia data and assumed some parameters of *M. genitalium*. The confidence intervals for those parameters of *M. genitalium* models cannot be obtained from published literature was estimated based on assumptions of binomial probability distributions ^2-4^ (Please see Table S2)

Table S2. *Mycoplasma genitalium* model inputs

| **Parameters** | **Value (uncertainty bounds)** | **References/Remarks** |
| --- | --- | --- |
| %, Consistent condom usage in anal sex in past 12 months | 46.90(34.50- 59.30) | ^5^ |
| %, Condom efficacy in preventing transmission | 87.50(80.00-95.00) | ^5^ |
| days, Frequency of kissing | 6.31(0.00-13.12) | ^5^ |
| days, Frequency of oral sex | 13.53(0.00-28.11) | ^5^ |
| days, Frequency of rimming | 38.57(0.00-80.15) | ^5^ |
| days, Frequency of anal sex | 26.44(0.00-54.94) | ^5^ |
| weeks, Infection duration of *Mycoplasma genitalium* at the throat (asymptomatic infection) | 95.29(24.87-245.44) | ^6,7^ |
| weeks, Infection duration of *Mycoplasma genitalium* at urethra (symptomatic infection) | 1.57(1.00-2.00) | Footnote *a*, ^8^ |
| weeks, Infection duration of *Mycoplasma genitalium* at urethra (asymptomatic infection) | 57.72(19.60-158.08) | Footnote *b*, ^9^ |
| %, Proportion of urethral *Mycoplasma genitalium* infections that are asymptomatic | 79.17(57.30-92.07) | Footnote *c*, ^10^ |
| %, Proportion of anal *Mycoplasma genitalium* infections that are asymptomatic | 93.33(80.68-98.26) | Footnote *c*, ^10^ |
| weeks, Infection duration at anorectum (symptomatic infection) | 1.57(1.00-2.00) | Footnote *a*, ^8^ |
| weeks, Infection duration of *Mycoplasma genitalium* at the anorectum (asymptomatic infection) | 82.68(50.02-134.16) | Footnote *b*, ^9^ |

**Footnote**:

1. The duration time is estimated by the time between the first symptom and treatment. Among Men, the time was 11 Days=1.57 weeks, range (7-14) days.
2. Estimated value from gonorrhoea and chlamydia infection. Mean urethral infection duration was 57.72[19.60-158.08] weeks. The value of infection duration of *Mycoplasma genitalium* at the anus was estimated from gonorrhoea and chlamydia infection.
3. Proportion of urethral *Mycoplasma genitalium* infections that are asymptomatic was 19/24, proportion of anal *Mycoplasma genitalium* infections that are asymptomatic was 42/45.

**Site-Specific infection data**

**Summary of anatomical site-specific infection prevalence data on *Mycoplasma genitalium***

One survey data with multi-site infection of *M. genitalium* prevalence among 1,011 asymptomatic MSM attending Melbourne Sexual Health Centre (MSHC) during 2016–2017 reported oropharyngeal infection ^11^. Only men with anorectal *M. genitalium* were tested for oropharyngeal *M. genitalium* in this study. The proportion of MSM who had multi-site infections with *M. genitalium* was 2.97% (3/101). The second study with multi-site infection data of *M. genitalium* prevalence among 508 MSM attending the Western Sydney Sexual Health Centre (WSSHC) from February to May 2017 did not report any oropharyngeal infections ^10^. The proportion of MSM who had multi-site infections with *M. genitalium* was 1.47% (1/68).The third study with multi-site infection data of *M. genitalium* prevalence among 521 MSM attending six male-only saunas in Melbourne was conducted between October 2001 and September 2002, and it did not report any oropharyngeal infections either ^12^. The proportion of MSM who had multi-site infections with *M. genitalium* was 0/11=0.00%.

Table S3. The anatomical site-specific infection prevalence of *Mycoplasma genitalium* in MSM

|  | Oropharynx alone | | Urethral alone | | Rectal only | | Oropharynx and urethra | | Oropharynx and anorectum | | Urethra and anorectum | | All three sites | |
| --- | --- | --- | --- | --- | --- | --- | --- | --- | --- | --- | --- | --- | --- | --- |
|  | Cases/  MSM tested | Footnote a, %, 95%CI | Cases/  MSM tested | %,95%CI | Cases/  MSM tested | %,95%CI | Cases/  MSM tested | %, 95%CI | Cases/  MSM tested | %, 95%CI | Cases/  MSM tested | %, 95%CI | Cases/  MSM tested | %,95%CI |
| Read^11^ | 1/54 | 1.9%  [0.3-9.8] | 27/1007 | 2.6%  [1.9-3.9] | 70/1005 | 7.0%  [5.6-8.7] | 0/54 | 0.0%  [0.0-6.6] | 1/54 | 1.9%  [0.3-9.8] | 2/1001 | 0.2%  [0.0-0.7] | 0/54 | 0.0%  [0.0-6.6] |
| Couldwell^10^ | 0/508 | 0.0%  [0.0-0.8] | 23/508 | 4.5%  [3.0-6.7] | 44/505 | 8.7%  [6.6-11.5] | 0/508 | 0.0%  [0.0-0.8] | 0/508 | 0.0%  [0.0-0.8] | 1/505 | 0.0%  [0.0-0.8] | 0/505 | 0.0%  [0.0-0.8] |
| Catriona^12^ | 0/515 | 0.0%  [0.0-0.7] | 3/510 | 0.59%  [0.2-1.7] | 8/497 | 1.6%  [0.8-3.1] | 0/510 | 0.0%  [0.0-0.8] | 0/497 | 0.0%  [0.0-0.8] | 0/497 | 0.0%  [0.0-0.8] | 0/497 | 0.0%  [0.0-0.8] |
| Weighted estimates from the above three datasets | 1/1077 | 0.1%  [0.0-0.5] | 53/2025 | 2.6%  [2.0-3.4] | 122/2007 | 6.1%  [5.1-7.2] | 0/1072 | 0.0%  [0.0-0.4] | 1/1059 | 0.1%  [0.0-0.5] | 3/2003 | 0.2%  [0.1-0.4] | 0/1056 | 0.0%  [0.0-0.4] |

Footnote

Prevalence was estimated based on assumptions of binomial probability distributions ^2-4^.

**Model calibration procedures**

The parameters of *M. genitalium* models were sampled within the confidence interval by the Latin hypercube sampling method and repeated 300 times. We measured the calibration error by calculating the sum of the squared estimate of errors. According to calibration error to select ‘optimal runs’, we sorted the simulation outputs in descending order, and 10% of 300 simulations were regarded as the calibrated model estimates to the epidemic trend and used to generate the *M. genitalium* model outputs with 95% confidence intervals. We used the ‘trust-region-reflective’ method for optimisation of a nonlinear function based on each selected set of parameters of *M. genitalium* model fittings for the weighted prevalence data were conducted using the built-in MATLAB least-squares fitting routine fmincon in the optimisation toolbox ^13^. We compared *M. genitalium* models using the minimal of sum squared errors between the empirical multisite infections data and the corresponding calibration results.

**Model 1 (Anal sex and oral sex) calibration**

To parameterise this model, 17 parameters values and 95% confidence intervals are needed (Table S3, S4): 2 parameters related to condom (consistent condom usage, and condom efficacy), 2 parameters related to sexual practices (Frequency of oral sex, and Frequency of anal sex) , 6 parameters related to the *Mycoplasma genitalium* infection recovery process (Infection duration of *Mycoplasma genitalium* at the throat (asymptomatic infection), Infection duration of *Mycoplasma genitalium* at urethral (symptomatic infection), Infection duration of *Mycoplasma genitalium* at urethral (asymptomatic infection), Proportion of urethral *Mycoplasma genitalium* infections that are asymptomatic, Proportion of anal *Mycoplasma genitalium* infections that are asymptomatic, and Infection duration of *Mycoplasma genitalium* at the anus), and 7 site-specific *Mycoplasma genitalium* infection prevalence parameters (oropharynx only, urethral only, rectal only, oropharynx and urethra both, oropharynx and rectum both, urethra and rectum both, and infection at all three sites).

**Model 2 (Oral sex and anal sex and rimming) calibration**

To parameterise this model, 18 parameters values and 95% confidence intervals are needed (Table S3, S4): 2 parameters related to condom (consistent condom usage, condom efficacy), 3 parameters related to sexual practices (Frequency of oral sex, Frequency of anal sex, and Frequency of rimming) , 6 parameters related to the *Mycoplasma genitalium* infection recovery process (Infection duration of *Mycoplasma genitalium* at the throat (asymptomatic infection), Infection duration of *Mycoplasma genitalium* at urethral (symptomatic infection), Infection duration of *Mycoplasma genitalium* at urethral (asymptomatic infection), Proportion of urethral *Mycoplasma genitalium* infections that are asymptomatic, Proportion of anal *Mycoplasma genitalium* infections that are asymptomatic, Infection duration of *Mycoplasma genitalium* at the anus), and 7 site-specific *Mycoplasma genitalium* infection prevalence parameters (oropharynx only, urethral only, rectal only, oropharynx and urethra both, oropharynx and rectum both, urethra and rectum both, and infection at all three sites).

**Model 3 (Oral sex and anal sex and kissing) calibration**

To parameterise this model, 18 parameters values and 95% confidence intervals are needed (Table S3, S4): 2 parameters related to condom (consistent condom usage, condom efficacy), 3 parameters related to sexual practices (Frequency of oral sex, Frequency of anal sex, and Frequency of kissing) , 6 parameters related to the *Mycoplasma genitalium* infection recovery process (Infection duration of *Mycoplasma genitalium* at the throat (asymptomatic infection), Infection duration of *Mycoplasma genitalium* at urethral (symptomatic infection), Infection duration of *Mycoplasma genitalium* at urethral (asymptomatic infection), Proportion of urethral *Mycoplasma genitalium* infections that are asymptomatic, Proportion of anal *Mycoplasma genitalium* infections that are asymptomatic, Infection duration of *Mycoplasma genitalium* at the anus), and 7 site-specific *Mycoplasma genitalium* infection prevalence parameters (oropharynx only, urethral only, rectal only, oropharynx and urethra both, oropharynx and rectum both, urethra and rectum both, infection at all three sites).

**Model 4 (Oral sex and anal sex and rimming and kissing) calibration**

To parameterise this model, 19 parameters values and 95% confidence intervals are needed (Table S3, S4): 2 parameters related to condom (consistent condom usage, condom efficacy), 4 parameters related to sexual practices (Frequency of oral sex, Frequency of anal sex, Frequency of rimming, and Frequency of kissing) , 6 parameters related to the *Mycoplasma genitalium* infection recovery process (Infection duration of *Mycoplasma genitalium* at the throat (asymptomatic infection), Infection duration of *Mycoplasma genitalium* at urethral (symptomatic infection), and Infection duration of *Mycoplasma genitalium* at urethral (asymptomatic infection), Proportion of urethral *Mycoplasma genitalium* infections that are asymptomatic, Proportion of anal *Mycoplasma genitalium* infections that are asymptomatic, Infection duration of *Mycoplasma genitalium* at the anus), and 7 site-specific *Mycoplasma genitalium* infection prevalence parameters (oropharynx only, urethral only, rectal only, oropharynx and urethra both, oropharynx and rectum both, urethra and rectum both, and infection at all three sites).

**References**

1 Birger, R. *et al.* Should we screen for the sexually-transmitted infection *Mycoplasma genitalium*? Evidence synthesis using a transmission-dynamic model. *Scientific reports* **7**, 16162, doi:10.1038/s41598-017-16302-8 (2017).

2 Newcombe, R. G. Two-sided confidence intervals for the single proportion: comparison of seven methods. *Stat Med* **17**, 857-872 (1998).

3 Hu, F. B. Diet and exercise for new-onset type 2 diabetes? *Lancet* **378**, 101-102, doi:10.1016/s0140-6736(11)60692-2 (2011).

4 Wilson, E. B. Probable Inference, the Law of Succession, and Statistical Inference. *Journal of the American Statistical Association* **22**, 209-212, doi:10.1080/01621459.1927.10502953 (1927).

5 Zhang, L. *et al.* Neisseria gonorrhoeae Transmission Among Men Who Have Sex With Men: An Anatomical Site-Specific Mathematical Model Evaluating the Potential Preventive Impact of Mouthwash. *Sex Transm Dis* **44**, 586-592, doi:10.1097/olq.0000000000000661 (2017).

6 Templeton, D. J. *et al.* Prevalence, incidence and risk factors for pharyngeal chlamydia in the community based Health in Men (HIM) cohort of homosexual men in Sydney, Australia. *Sex Transm Infect* **84**, 361-363, doi:10.1136/sti.2008.032037 (2008).

7 Chow, E. P. *et al.* Duration of gonorrhoea and chlamydia infection at the pharynx and rectum among men who have sex with men: a systematic review. *Sex Health* **13**, 199-204, doi:10.1071/SH15175 (2016).

8 Andersen, B. *et al.* Prediction of costs, effectiveness, and disease control of a population-based program using home sampling for diagnosis of urogenital Chlamydia trachomatis Infections. *Sex Transm Dis* **33**, 407-415, doi:10.1097/01.olq.0000200609.77577.3f (2006).

9 Jin, F. *et al.* Incidence and risk factors for urethral and anal gonorrhoea and chlamydia in a cohort of HIV-negative homosexual men: the Health in Men Study. *Sex Transm Infect* **83**, 113-119, doi:10.1136/sti.2006.021915 (2007).

10 Couldwell, D. L. *et al.* *Mycoplasma genitalium*: high prevalence of resistance to macrolides and frequent anorectal infection in men who have sex with men in western Sydney. *Sexually transmitted infections* **94**, 406-410, doi:10.1136/sextrans-2017-053480 (2018).

11 Read, T. R. H. *et al.* Symptoms, Sites, and Significance of *Mycoplasma genitalium* in Men Who Have Sex with Men. *Emerg Infect Dis* **25**, 719-727, doi:10.3201/eid2504.181258 (2019).

12 Bradshaw, C. S. *et al.* *Mycoplasma genitalium* in men who have sex with men at male-only saunas. *Sex Transm Infect* **85**, 432-435, doi:10.1136/sti.2008.035535 (2009).

13 Onwubu, S. C., Mdluli, P. S., Singh, S. & Collins, O. C. The Application of the Logistic Equation Model to Predict the Remineralisation Characteristics of Desensitizing Paste. *Int J Dent* **2019**, 7528154, doi:10.1155/2019/7528154 (2019).
